# Meeting report: Identifying practical applications of ontologies for biodiversity informatics

**DOI:** 10.1186/s40793-015-0014-0

**Published:** 2015-05-17

**Authors:** John Deck, Robert Guralnick, Ramona Walls, Stanley Blum, Melissa Haendel, Andréa Matsunaga, John Wieczorek

**Affiliations:** 1 1007 Valley Life Sciences Building, University of California at Berkeley, Berkeley 97420, CA, USA; 2 Florida Museum of Natural History, University of Florida at Gainesville, Gainesville 32611-2710, FL, USA; 3 The iPlant Collaborative, University of Arizona, Thomas J. Keating Bioresearch Building, 1657 East Helen St., Tucson 85721, AZ, USA; 4 Institute for Biodiversity Science and Sustainability, California Academy of Sciences, 55 Music Concourse Drive, San Francisco 94118, CA, USA; 5 Department of Medical Informatics and Epidemiology, Oregon Health & Science University, Portland, USA; 6 University of Florida, Gainesville 32611, FL, USA; 7 3101 Valley Life Sciences Building, University of California at Berkeley, Berkeley 97420, CA, USA

**Keywords:** Ontology, Biodiversity, Population, Community, Darwin core, OWL, RDF, Microbial ecology, Sequencing

## Abstract

This report describes the outcomes of a recent workshop, building on a series of workshops from the last three years with the goal if integrating genomics and biodiversity research, with a more specific goal here to express terms in Darwin Core and Audubon Core, where class constructs have been historically underspecified, into a Biological Collections Ontology (BCO) framework. For the purposes of this workshop, the BCO provided the context for fully defining classes as well as object and data properties, including domain and range information, for both the Darwin Core and Audubon Core. In addition, the workshop participants reviewed technical specifications and approaches for annotating instance data with BCO terms. Finally, we laid out proposed activities for the next 3 to 18 months to continue this work.

## Introduction

Starting in 2009, the Research Coordination Network for the Genomic Standards Consortium has promoted its mission of integrating information on genomic and biodiversity resources. Directed activities since 2009 have focused on a series of workshops and collaborative programming or coding sessions (“hackathons”) with increasingly focused outcomes: vocabulary refinement for the Minimum information about any (x) sequence, representing genomic information standards, and Darwin Core, representing biodiversity information standards, expressing biodiversity concepts using the Basic Formal Ontology [1], introducing the concept of a material sample into the Darwin Core standard [2], conceiving and establishing the Biological Collections Ontology [3], and more recently, identifying practical applications of BCO [4]. Other specific outcomes of these efforts have included the creation of an RDF version of the MIxS standard [5], and numerous term refinements for both the Darwin Core and MIxS vocabularies. Ultimately, our goal is to enable the integration of large and heterogeneous biodiversity datasets using BCO annotations and associated semantic web-oriented tools. Details on specific use cases for the BCO have been described in previous workshops and work on the BCO [3],[4].

The Eugene meeting ran from August 23 – 25, 2014 with a specific goal of advancing ongoing work in representing Darwin Core and Audubon Core terms in BCO and proposing mechanisms for expressing publicly available data with BCO annotations. While both areas of activity had their antecedents in recently completed workshops, the work had yet to be fully completed. Since the BCO has its roots in the Basic Formal Ontology, we have chosen to continue focusing our term mapping, for now, on BFO classes for logical consistency. Exemplar data sets used to test the efficacy of translating DwC to BCO were drawn from VertNet [6] and iDigBio [7]. Below we discuss outcomes from this workshop, focusing especially on the key outcome of expressing terms in Darwin Core and Audubon Core, where class constructs have been historically underspecified, into a BCO framework, where we strive to fully define classes as well as object and data properties, including domain and range information.

## Activities and analysis

The meeting consisted of a small group of individuals (see Attendees), with a focus on reviewing recent work, actively completing outstanding tasks, and planning for future work. The key outcome was to complete mappings between BCO with DwC, including importing DwC basisOfRecord terms, which are essentially the rdfs:classes from DwC, into BCO and assigning domains for all DwC properties to BCO classes and ranges to ontology classes or literals. Some properties were qualified based on the value of other properties. For example, lifeStage is assigned the domain caro:organism when the individualCount is 1, but its domain is dwc:occurrence when the individualCount is greater than 1. The following sections describe the mapping process for the specific topics focused on during this meeting.

### Basis of record

The DwC property basisOfRecord [8] is defined as “The specific nature of the data record - a subtype of the dcterms:type [9]. Recommended best practice is to use a controlled vocabulary such as the Darwin Core Type Vocabulary^a^. The term basisOfRecord plays a role similar to, but more specific than that of dcterms:type, which expresses the Dublin Core resource type (PhysicalObject, Event, StillImage, MovingImage, Sound, Text, etc.). The value of basisOfRecord is recommended to be drawn from the Darwin Core Type controlled vocabulary, but this vocabulary has serious limitations. First, there are missing logical relations between the terms (e.g., both HumanObservation and MachineObservation are types of observations, but observation is not defined anywhere) that cannot be understood without a machine readable specification of those relations. Second, the current vocabulary is limited in its expressivity by only including high-level terms. With the need to describe and exchange complex types of biodiversity data comes a need to provide more specific metadata on the type of record (e.g., was a machine observation made by a camera trap or an audio recorder?). Finally, the Darwin Core type vocabulary is difficult to adapt to new uses, because it is the only controlled vocabulary for which the governance falls under the rules of the Darwin Core Namespace policy [10], under which new terms require a lengthy and rigorous community consultation process. In contrast, new terms can be added to most ontologies relatively easily, providing a test-bed for the utility and applicability of a term before it is proposed as part of the Darwin Core standard.

At the hackathon, logical definitions were created for the DwC type vocabulary, which was then mapped to BCO by determining where to position each DwC class in the BCO hierarchy. In addition, an initial proposal was drafted recommending that the controlled vocabulary for basisOfRecord be drawn from a subset of BCO rather than from the Darwin Core Type vocabulary, with maintenance by BCO editors and governance continuing under TDWG. This proposal is proposed to be considered further in the Darwin Core and Genomic Biodiversity Working Group sessions at the annual TDWG meeting in October, 2014 in Jönköping, Sweden. The following sections provide some specific examples of DwC classes mapped to BCO.

### Representing location in BCO

**Figure 1:**
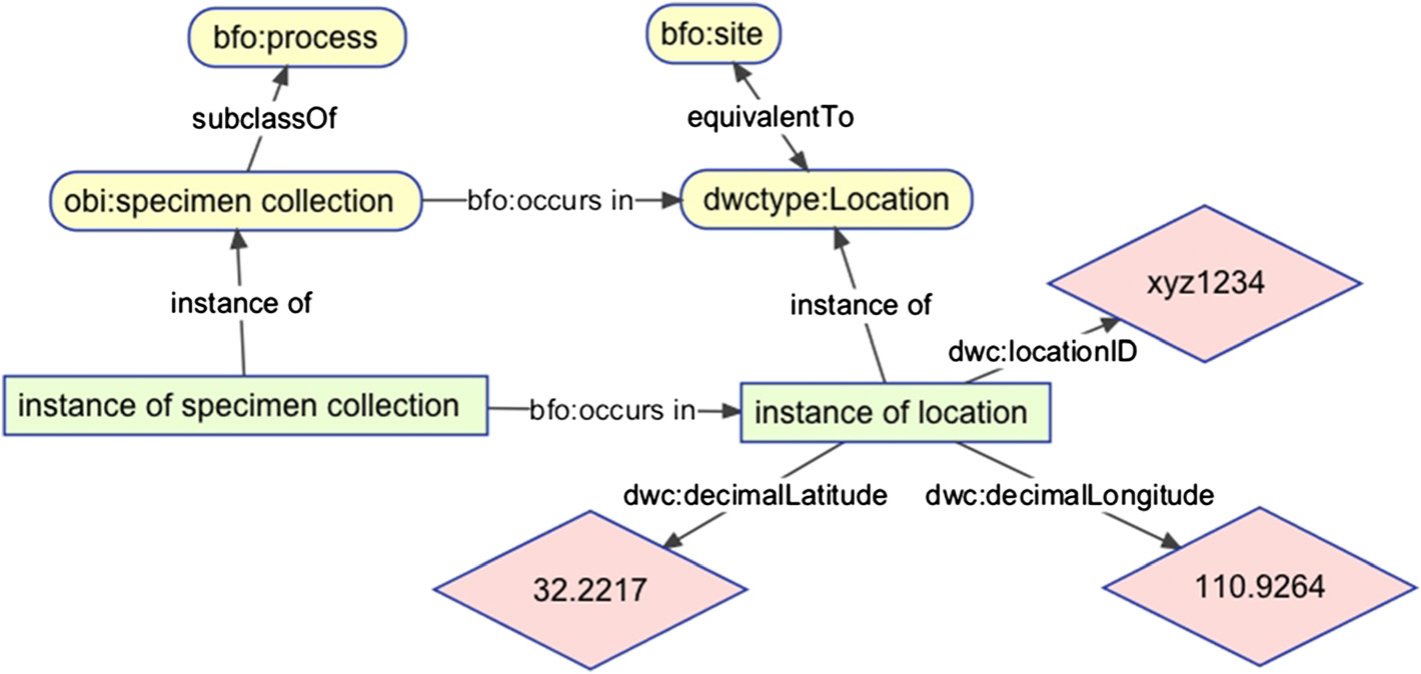
A graphical representation of how Location from Darwin Core fits into the Biological Collections Ontology. Yellow rounded rectangles are classes, green rectangles are instances, pink diamonds are literals. Ontologies are abbreviated as “bfo” for Basic Formal Ontology, “obi” for Ontology for Biomedical Investigations, and “dwctype” for Darwin Core Type Vocabulary, and “dwc” for Darwin Core.

The DwC Type class Location is defined (using a comment in Darwin Core) as “A resource describing an instance of the Location class” where the Location class refers to the Dublin Core term dcterms:Location [11], adopted for use in Darwin Core with the amended definition “A spatial region or named place. For Darwin Core, a set of terms describing a place, whether named or not.” In BCO, we interpret Location as equivalent to the class ‘site’ from BFO (i.e. bfo:site), which is elucidated as “a three dimensional immaterial entity that is (partially or wholly) bounded by a material entity or it is a three-dimensional immaterial part thereof” [12]. Thus, dcterms:Location or bfo:site can refer to either relative locations, such as part of an organism, or geographic locations (which are also relative to the earth). We added an axiom to BCO that dwctype:Location is equivalent to bfo:site. The key point for describing DwC data is that each instance of Event [13] -- for example, some instance of specimen collection -- occurs in some instance of “Location” at some time. This allows us to specify dwctype:Location as the domain of a number of DwC properties, such as decimalLatitude, decimalLongitude, and locationID (Figure 1), and use simple SPARQL queries to search for collection events that took place at particular locations, such as bounding box specified by geographic coordinates.

### Representing taxon and identification in BCO

We chose to map the DwC classes Taxon and Identification together because of their logical interdependence. The DwC Type class Taxon is defined (using a comment in Darwin Core) as “A resource describing an instance of the Taxon class”, where the “Taxon class” refers to the Darwin Core Taxon class [14] with the additional definition “The category of information pertaining to taxonomic names, taxon name usages, or taxon concepts.”

**Figure 2:**
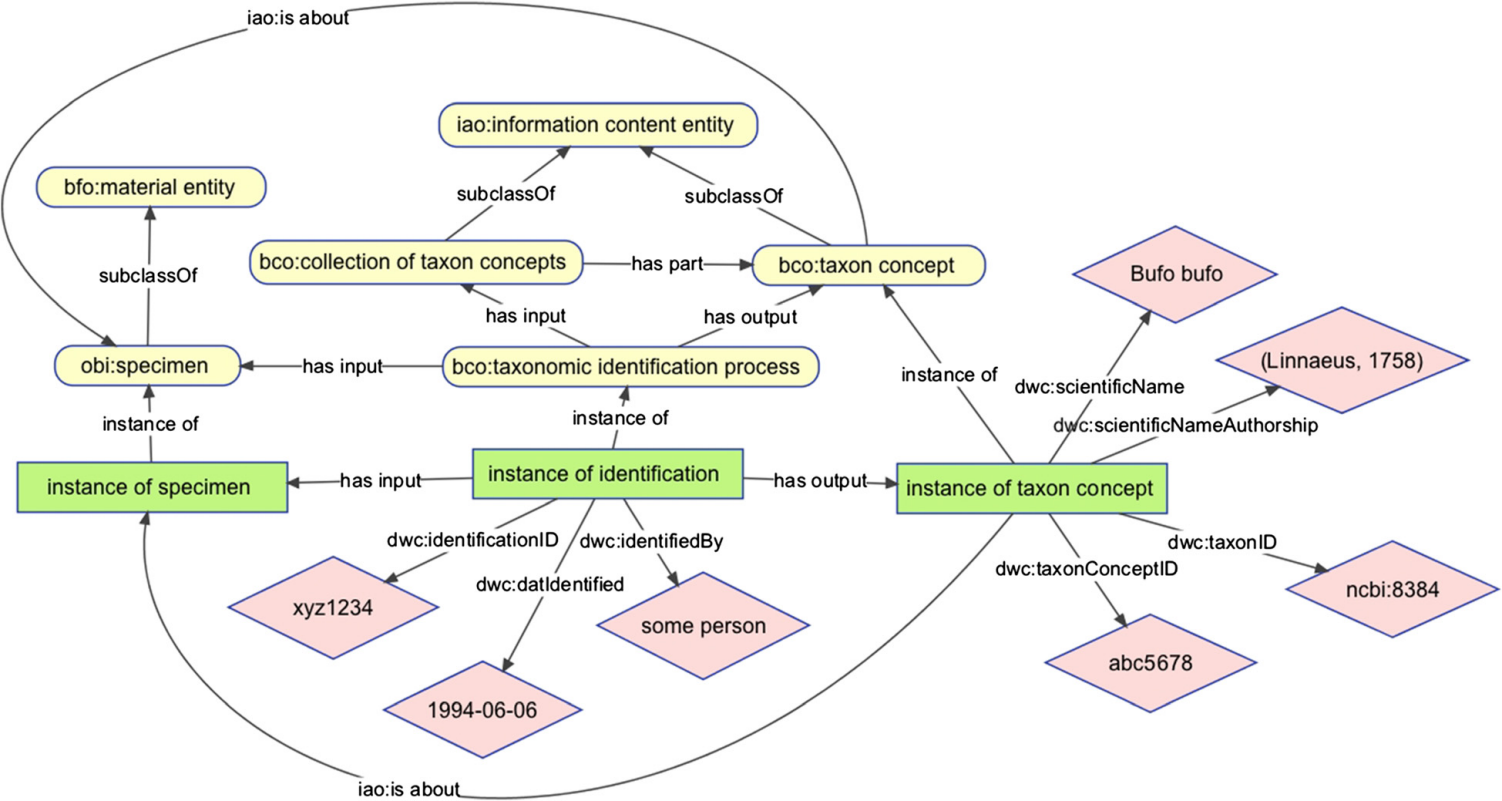
An ontological representation of the identification process in the Biological Collections Ontology. Colors and shapes are as in Figure 1. Ontologies are abbreviated as “bfo” for Basic Formal Ontology, “obi” for Ontology for Biomedical Investigations, “iao” for Information Artifact Ontology, “bco” for Biological Collections Ontology, and “dwc” for Darwin Core.

The DwC Identification class [15] has no equivalent in the DwC Type vocabulary, and is defined as “The category of information pertaining to taxonomic determinations (the assignment of a scientific name)”. The textual definitions for Taxon and Identification do not provide much of a basis for logical definition in an ontology, so we felt it was better to create new classes in BCO to represent the identification process and the taxonomic concepts or names that are the inputs to and the outputs from of that process (Figure 2). In our model, a taxonomic identification process takes as input a specimen (this could also be a live organism *in situ*) and a collection of taxonomic concepts and has as output a taxon concept that is associated with (is about) the specimen. This model allows us to use reasoning to track the provenance of taxonomic identification processes and link specimens to one or more taxon concepts. We recognize that in many cases the only recorded information during taxonomic identifications is a taxon name, not a concept, but the process almost always involves concepts whether implicit or explicit.

### Representing geological context in BCO

We also briefly discussed the DwC class “GeologicalContext” [16] and decided that the modeling needed to correctly represent information pertaining to this Darwin Core class was beyond the immediate scope of the BCO. We instead agreed to seek input from geology domain experts in the future on their modeling of chronostratigraphy, lithostratigraphy, and biostratigraphy. These can serve as the basis for application profiles for representing paleontological data that draws from BCO and other source ontologies.

### Representing Audubon core in BCO

The iDigBio dataset contains information about media associated with specimens (images, videos, audio, 3D models, etc.), capturing important features of the organism, and sometimes serving as a voucher for the actual specimen. This type of information is captured using the Audubon Core vocabulary, and we have begun mapping these terms to BCO. Since Audubon Core adopts some DwC terms, those were mapped as described previously in this report. The additional terms were mainly mapped into the media domain and these represent attributes of the media to identify, access and provide proper attribute to the media (e.g., identifier, accessURI, rights, webStatement). The term ac:associatedSpecimenReference is important as it creates the linkage between the media object and specimens, and should have as its range the URI denoting the Specimen.

### Data set mapping

We explored mapping VertNet and iDigBio data sets to BCO. Several VertNet Darwin Core Archives (DwCAs) [17] were identified as good candidates for a test-bed converter to be coded as BCO. Discussion took place on the practicality of processing DwCAs to generate RDF triple output, using BCO term annotations. This process was similar to efforts from the February 2014 Tucson BCO meeting [4], where we analyzed and converted to RDF triples various spreadsheets representing barcoding and soil sampling. The process was also similar to the one in the March 2014 GSC Oxford meeting where we did the same for Ocean Sampling Day [18] spreadsheets. Based on the details of harmonizing DwC with BCO, we decided the process of triplifying (e.g. creating RDF triple output using the Triplifer toolkit [19]) DwCAs annotated with BCO terms was more complex than the spreadsheet generation process and deserved further work, beyond the scope of this particular workshop.

The final day of the meeting we had an extensive discussion on available tools and methods being used by the Monarch Initiative and eagle-i projects [20] with Principal Investigator Melissa Haendel. We talked about the use of both WebKarma [21] and RightField [22], being explored for use in the Monarch Initiative for annotating data using ontologies. Also discussed were tools for creating and managing instance identifiers, including the Resource Identification Initiative [23], EZID [24], and BCID [25].

## Products and conclusions

The meeting generated ideas for several products, to be finished in the near future.

### Darwin core type vocabulary as a subset of BCO

We proposed to change the recommended controlled vocabulary for basisOfRecord from the Darwin Core Type vocabulary to a subset of the Biological Collections Ontology. Existing Darwin Core Type vocabulary terms for basisOfRecord would remain with their existing URIs, but would be imported into the BCO, where additional logical axioms would be specified.

### DwCA to BCO converter

We built a proposal for creating an application library for converting Darwin Core Archives to BCO annotated triples. While the simplicity of DwCAs for publishing biodiversity data has been a significant reason for the rise in popularity of this exchange format, mapping to an ontology requires the rigorous definition of relations that are not explicit in the original data. Thus, the coding is somewhat complicated and requires specialized logic to decipher and apply the relevant BCO domains of DwC terms. Thus, we propose to build a Java-based command-line tool and REST web-service for converting archives to BCO-coded RDF triples.

### TDWG interactions

Meeting participants discussed interaction possibilities for the TDWG 2014 meeting, including participating in the scheduled sampling symposium, proposing a BCO task group as part of the GBWG interest group, and putting together a presentation on BCO. TDWG represents a key and continuing organization for presenting results and working towards further community involvement in standards growth related to BCO and allied ontologies.

## Future work

Another meeting outcome was to establish a roadmap of future work, extending the efforts broadly coupled to the RCN4GSC project with contributions from key partner organizations and projects such as BiSciCol [26], iPlant [27], i3B [28], NEON [29], and VertNet [6]. The trajectory of our work has been to clarify concepts and terms, build a bio-collections ontology, and translate data from existing simple formats to new representations where reasoning is enabled. To that end, we established the following roadmap:

### 0-3 months

 Design a method for provenance tracking in VertNet. In the process of data publishing, data can undergo transformations from the original to something putatively improved, or easier to discover or use, with a Darwin Core Archive as the preferred format for publication. The idea behind provenance tracking is to capture the transformations as an extension to the published archive.

 Hold a meeting in Florence 11–12 Sep 2014 to a) create MIxS-like checklists [30] of Darwin Core records based on basisOfRecord [31], b) update MIxS as RDF to the most current version directly from the database where the terms are managed, and c) mark up DwC with MIxS extension Darwin Core archives from sample data sets from GBIF and VertNet.

 Make proposal for the basisOfRecord vocabulary to be represented as part of the ontology in BCO, presenting the results at TDWG 2014.

 Produce and disseminate prototype BCO triple data from VertNet and iDigBio experiments.

 Standardize resolver metadata response for RDF/XML accept header requests applied to specimen DOIs (and any other identifier) that is customized for specimen records (instead of using responses customized for published works).

### 3–9 Months

 Enable queries beginning with BCO and connecting to data stored as Darwin Core Archives:

 ○ First prototype of data conversion framework that links to ontologies for VertNet. Outcome: Build a prototype for querying Darwin Core records with terms from BCO

 ○ Mapping BCO to DarwinCore

 ○ Link to GenBank data and image data at iDigBio

 ○ Data handling task

### 9-18 months

 Generalize and extend 6 month deliverables. Starting point: Spreadsheet conversion tools from large-scale producers of biodiversity and biodiversity genomics data.

 Work towards a new Research Coordination Network proposal, that is focused on best practices across the currently emerging landscape, as Darwin Core, Audubon Core, ABCD, and other standards and new ontologies reach maturity. Managing and dealing with governance and the scope of proposed changes to these standards has exceeded the capacity of the volunteer efforts in the Biodiversity Information Standards (TDWG) organization.

### 18 months + (Long term thinking:)

 Build tools and methods to facilitate data generation and aggregation that enables full-database retrieval, analysis, and ultimately automated reasoning not predicated on the presumption of high data quality coming from each of the participating data sources.

### Workshop attendees

John Deck (lead organizer); Stan Blum; Tom Conlin; Rob Guralnick; Melissa Haendel; Andrea Matsunaga; Bob Robbins; Ramona Walls; John Wieczorek.

### Endnote

^a^While the terms in the Darwin Core type vocabulary have recently been removed in favor of equivalent terms in the normal Darwin Core namespace, we have chosen to retain references to the Darwin Core type vocabulary as it was still active at the time of this workshop. In addition, all web related references to the Darwin Core type vocabulary and related class definitions have since been removed from the web, hence, no references are given for these resources.

## Abbreviations

ABCD: Access to biological collections data

AC: Audubon core

BCO: Biological collections ontology

BFO: Basic formal ontology

CARO: Common anatomy reference ontology

DCTERMS: Dublin core terms

DOI: Digital object identifier

DwC: Darwin core

DwCA: Darwin core archive

DWCTYPE: Darwin core type vocabulary

GBIF: Global biodiversity information facility

iDigBio: Integrated digitized biocollections

MIxS: Minimum information about any (x) sequence

RCN4GSC: Research coordination network for the genomic standards consortium

RDF: Resource description framework

RDFS: RDF schema

REST: Representation state transfer

TDWG: Biodiversity information standards

URI: Uniform resource identifier

XML: Extensible markup language

## Competing interests

The authors declare that they have no competing interests.

## Authors’ contributions

All authors participated the workshop and contributed to the writing of this document. All authors read and approved the final manuscript.
